# Gene-environment interaction in functional hypothalamic amenorrhea

**DOI:** 10.3389/fendo.2024.1423898

**Published:** 2024-08-29

**Authors:** Federica Barbagallo, David Bosoni, Valeria Perone, Laura Cucinella, Davide Dealberti, Rossella Cannarella, Aldo E. Calogero, Rossella E. Nappi

**Affiliations:** ^1^ Department of Clinical and Experimental Medicine, University of Catania, Catania, Italy; ^2^ Department of Obstetrics and Gynecology, Azienda Ospedaliera Nazionale SS. Antonio e Biagio e Cesare Arrigo, Alessandria, Italy; ^3^ Department of Clinical, Surgical, Diagnostic and Pediatric Sciences, University of Pavia, Pavia, Italy; ^4^ Research Center for Reproductive Medicine, Gynecological Endocrinology and Menopause, IRCCS San Matteo Foundation, Pavia, Italy; ^5^ Glickman Urological and Kidney Institute, Cleveland Clinic, Cleveland, OH, United States

**Keywords:** functional hypothalamic amenorrhea (FHA), idiopathic hypogonadotropic hypogonadism, genetic susceptibility, epigenetic, stress, male equivalent of FHA

## Abstract

Functional hypothalamic amenorrhea (FHA) is a common cause of amenorrhea and chronic anovulation in adolescent girls and young women, diagnosed after excluding other organic causes. It is commonly associated with calorie restriction, excessive physical exercise, and psychosocial stress. These stressors alter the pulsatile secretion of gonadotropin-releasing hormone, leading to a chronic condition of hypoestrogenism and significant health consequences. Recent evidence has highlighted a genetic predisposition to FHA that could explain interindividual variability in stress response. Indeed, not all women experience FHA in response to stress. Rare variants in genes associated with idiopathic hypogonadotropic hypogonadism have been identified in women with FHA, suggesting that these mutations may contribute to an increased susceptibility of women to the trigger of stress exposure. FHA appears today as a complex disease resulting from the combination of genetic predisposition, environmental factors, and epigenetic changes. Furthermore, the genetic background of FHA allows for the hypothesis of a male counterpart. Despite the paucity of data, preliminary findings indicate that an equivalent condition of FHA exists in men, warranting further investigation. This narrative review aims to summarize the recent genetic evidence contributing to the pathophysiology of FHA and to raise awareness on a possible male counterpart.

## 1 Introduction

Functional hypothalamic amenorrhea (FHA) is a common cause of amenorrhea and chronic anovulation in adolescent girls and young women (1). FHA is responsible for approximately 20-35% of secondary amenorrhea in women of reproductive age and approximately 3% of primary amenorrhea (2), which is estimated to affect 17.4 million women worldwide (3).

FHA is defined as the absence of menses for at least 3 to 6 months, low or normal gonadotropin levels, and hypoestrogenism without organic causes. The term FHA is used to describe the lack of menstruation resulting from exposure to various types of stress, after excluding other etiologies of amenorrhea. It is commonly associated with three main stressful conditions: caloric restriction and weight loss, strenous physical exercise, and psychosocial stress, which may occur alone or, more often, in combination (1).

These stressor triggers reduce the pulsatile secretion of hypothalamic gonadotropin-releasing hormone (GnRH) (4), which impair the pulsatile release of luteinizing hormone (LH) resulting in decreased production of 17ß-estradiol in the ovary. Pulsatile GnRH secretion is influenced by a multitude of factors, hormonal and non-hormonal, which contribute to the complex pathophysiology of FHA, that is not yet fully understood (5).

Hypoestrogenism resulting from suppression of the hypothalamic-pituitary-ovarian (HPO) axis can impact the function of multiple systems including reproduction, bone, emotional, cognitive, and cardiovascular health (3, 6). Therefore, timely diagnosis and management of this condition are essential to prevent long-term consequences.

Recent evidence suggests a genetic predisposition to FHA (7–9). Rare variants of genes linked to the development and function of GnRH neurons have been identified in women with FHA (7, 8) and could explain interindividual variability in response to stressful events. Indeed, not all women experience FHA during stress exposure. Epigenetic changes could also compromise several pathways involved in the HPO axis, contributing to the onset of FHA (10). Furthermore, although little studied, a corresponding condition has also been described in men (11).

On this basis, this narrative review aims to summarize the recent genetic evidence contributing to the pathophysiology of FHA, while also raising awareness about the existence of a possible male counterpart.

## 2 Stressor triggers and their impact on the hypothalamic-pituitary-gonadal axis

In 1974, Frisch & McArthur proposed that a minimum level of body fat was necessary for ovulation and menstrual cycles in women (12). Early studies suggested that body fat percentage had to reach the “critical threshold” of 17% for menarche to occur and that secondary amenorrhea could be triggered if body fat percentage fell below 22% (12). However, subsequent studies have contradicted this hypothesis by showing that female athletes with low body fat have regular menses (13). Indeed, it is difficult to establish a body weight or fat cuf-off for having a regular menstrual cycle due to individual differences, and the change in physiological state appears to be more important than the absolute value (14). A condition of low energy availability can result from insufficient energy intake, as seen in eating disorders, or excessive energy expenditure, as seen with strenous physical exercise.

In 1992, the American College of Sports Medicine first coined the term “female athlete triad”, referring to a constellation of 3 clinical entities: menstrual dysfunction, low energy availability (with or without an eating disorder), and decreased bone mineral density (15). The prevalence of FHA in sportswomen is variable depending on the type of activity, duration, and intensity, with the highest prevalences reported in esthetic (such as cheerleading, diving, and gymnastics), endurance, and weight-class sports (16). When energy expenditure exceeds dietary energy intake due to intense training, the resulting macro- and micronutrients imbalance can alter the HPO axis function, contributing to the onset of FHA (17). This alteration in body composition translates into reduced adipokines production and an alteration in the levels of hormones that regulate appetite. Among these, leptin has a central role in the regulation of the HPO axis.

Leptin is a 16-kDa adipokine produced by adipose tissue. In addition to its well-known role in the regulation of food intake where it acts as a potent satiety factor, leptin may also have other functions. These include the regulation of reproduction, acting as both a central and peripheral factor. Low leptin levels are associated with GnRH suppression and leptin treatment has been shown to increase LH pulsatility in women with FHA (18). The effects of leptin on hypothalamic GnRH release are not direct since GnRH-secreting neurons do not express receptors for this adipokine. The central role of leptin in reproduction appears to be mediated by other peptides, including kisspeptin. Indeed, kisspeptin/neurokinin B/dynorphin neurons of the hypothalamic arcuate nucleus secrete kisspeptin, which acts on GnRH-secreting neurons by stimulating the release of GnRH (19). Several other factors, including neuropeptide Y, oxytocin, ghrelin, thyroid hormones, and insulin, interact with each other and play an important role in the pathophysiology of energy imbalance-related amenorrhea (14). Low levels of insulin (20) and triiodothyronine (21) characterize a state of negative energy balance and are often described in patients with FHA.

Psychological disorders are common in FHA patients and may themselves be a cause of FHA or synergize with metabolic stress (22). Psychosocial stressors are variable and depend on the individual’s perception (23). Patients with FHA have been demonstrated to cope less well with stress in their autonomic responses, with significantly higher increases in heart rate, systolic and diastolic blood pressure, and serum cortisol levels, in response to exposure to neuropsychological stress, compared to controls (24). Recent data indicate that FHA may contribute to preclinical cardiovascular dysfunction, not explained by hypoestrogenemia alone (25).

Stress, regardless of its type, activates the hypothalamic-pituitary-adrenal (HPA) axis and the autonomic nervous system, causing a constellation of neuroendocrine alterations (26). Both chronic and severe acute stressors can impact the function of the HPG axis. Stress and the resulting activation of the HPA axis impair HPG axis function, as reproduction is not a priority in the “fight or flight” response during stressful situations (27). Activation of the HPA axis is due to increased secretion of corticotropin-releasing hormone (CRH), adrenocorticotrophin (ACTH), cortisol, and endogenous opioids. Glucocorticoids (GLCs) interfere with reproductive function at both central and peripheral levels. Studies have shown that corticosterone, the main GLC in rodents, and dexamethasone can suppress GnRH release *in vitro*, confirming the central effect of GLCs (28). Furthermore, endogenous opioids also play a relevant role in inhibiting the pulsatile release of both GnRH and gonadotropins during stressful events (1) along with other neurotransmitters, including serotonin (29).

## 3 Inter-individual variability in the response to stress

Stressors are condition that threatens to alter or alters the homeostasis of an organism. As a result, a complex array of responses is activated to counterpoise the imbalance caused by stressful forces. Stress response mechanisms involve the endocrine, nervous, and immune systems, collectively referred as stress response effectors (30). FHA can be considered a natural protective mechanism in women against stressful events, which lead to the temporary suppression of gonadal function when physical conditions are not suitable to support a pregnancy (10). The onset times of menstrual irregularities following exposure to a stressful factor are variable; generally, the alterations begin with oligomenorrhea and then evolve into amenorrhea (1). Although the stress triggers leading to FHA are well documented, there is limited understanding of individual differences in how people respond to stress and the resulting impact on the HPO axis (10). Notably, not all women develop FHA under stressful circumstances (10). At the same time, the timing of the return of menses also varies greatly and to date we cannot predict whether the menstrual cycle resumes after the restoration of normal weight and energy balance in individual women (31). Recovery times appear to be influenced by the initial cause of FHA, with female athletes having a quicker recovery period than women with eating disorders (32, 33). As we will discuss in the following paragraphs, a genetic vulnerability, arising from rare or polymorphic variants of genes that control the development, homing, and function of GnRH neurons, could explain the variable impact of stress on FHA in individual women (7–10). Epigenetic modifications could also play a role and could potentially influence individual susceptibility to amenorrhea following stressful events or recovery times after experiencing FHA.

## 4 Genetics of functional hypothalamic amenorrhea

### 4.1 Idiopathic hypogonadotropic hypogonadism: genotype vs. phenotype

Idiopathic hypogonadotropic hypogonadism (IHH) is caused by defects in hypothalamic GnRH secretion or by defects in the action of GnRH on the pituitary gland, failing puberty onset or hypogonadism that occurs after the onset of puberty (34). IHH is a rare, genetically heterogenous disorder, with a prevalence of 1:125,000 in females and 1:30,000 in males (35). This form of hypogonadism is classified into IHH associated with hyposmia/anosmia, also known as Kallmann syndrome (KS) and normosmic IHH (nIHH). The reduction of smell in KS can be explained by the common embryogenic origin of GnRH-secreting neurons and olfactory neurons. Indeed, during embryogenesis, GnRH neurons migrate from the olfactory epithelium to their final position in the hypothalamus. With puberty, these neurons begin to secrete GnRH in a pulsatile pattern and this is the key stimulator of LH and FSH secretion. IHH is caused by numerous and rare sequence variants in several genes that encode proteins essential for the development, differentiation, and homing of GnRH-secreting neurons and for the secretion and action of GnRH (36).

To date, approximately 40% of patients with IHH have an identifiable genetic mutation (37). The first gene believed to be responsible for KS is the *KAL1* gene, mapping on the distal portion of the X chromosome (Xp22.3). *KAL1* encodes for the protein anosmin 1, which drives neural cell adhesion and axonal migration. Its mutation causes a migratory defect and therefore a failed or incorrect homing of GnRH-secreting neurons (38). Since then, understanding of the genetic basis of IHH has expanded significantly. This progress has been aided by notable technological advances, particularly the introduction of next-generation sequencing (NGS) methods. Numerous other genes have been identified, some primarily cause KS, others cause nIHH, while others cause either one or the other form (39). In addition to *KAL1*, other genes can disrupt the development and migration of GnRH neurons. These include *fibroblast growth factor receptor 1 (FGFR1), fibroblast growth factor 8, prokineticin 2 (PROK2), and PROK receptor 2 (PROKR2)* genes. Mutations on *GnRH receptor* (*GnRHR*) gene have also been identified. Other genes that interfere with the neuroendocrine physiology of normal GnRH secretion include *k*isspeptin 1 *(KISS1*), *Kiss1 receptor, leptin (LEP), LEP receptor (LEPR*)*, tachykinin 3 (TAC3), and TAC3 receptor 3*, which encode for neurokinin B and its receptor, respectively (39). Many of these genes have been shown to interact oligogenically, and most of them act in both neurodevelopmental and neuroendocrine pathways. A high genetic variability can be explained by an incomplete penetrance in variable expressivity that characterizes this disease (39).

Previous studies have also investigated the phenotype-genotype correlation of *GnRHR* gene mutations in male and female patients with different forms of GnRH deficiency (40–42). *GnRHR* mutations were first described by de Roux and colleagues in 1997 (43). Since then, more than 20 mutations in the coding sequence of the *GnRHR* gene have been reported in patients with sporadic or familial forms of IHH.

In 2012, Gianetti and coworkers screened a cohort of patients with different forms of GnRH deficiency, including adult-onset IHH (280 males and 95 females), KS (272 males and 88 females), FHA (77 females), and constitutional delay of puberty (29 males and 22 females) for *GnRHR* gene mutations (40). Among the 863 probands, the 70 mutation-carrying patients were divided into 4 groups (G1-G4) based on the severity of the mutations (complete or partial loss of function) and the number of allelic mutations (monoallelic or biallelic).

Then, the authors correlated the identified genotypic classes (G1-G4) to patient clinical phenotypes (40). Interestingly, in groups G1-G3, the patient phenotypes were consistent with severe GnRH deficiency. In contrast, G4 patients, with only monoallelic mutations, demonstrated a greater diversity of clinical phenotypes, ranging from severe GnRH deficiency such as KS and IHH to mild GnRH deficiency such as FHA, adult-onset IHH, or constitutional delay of puberty, to normal GnRH function. Therefore, these data (40) indicate that in patients with biallelic mutations of the *GnRHR* gene it is possible to identify a correlation between genotype and phenotype severity. In contrast, for patients with monoallelic *GnRHR* mutations, this correlation was not observed. Therefore, it can be hypothesized that other genetic or non-genetic factors (such as environmental or psychosocial stress) in combination with the mutated *GnRHR* allele contribute to decreased HPO axis function. This hypothesis cannot be excluded for biallelic mutations even if there seems to be a clear correlation between genotype and phenotype, as stated. Similarly, Beneduzzi and colleagues reported a relatively good genotype-phenotype correlation in male patients with IHH and *GnRHR* mutations (41).

### 4.2 Genetic overlap between functional hypothalamic amenorrhea and idiopathic hypogonadotropic hypogonadism

For the first time, in 2011, Caronia and colleagues showed that rare variants of IHH-associated genes can be found in patients with FHA, suggesting that these mutations may contribute to an increase in women’s susceptibility to stressors (7). In their study, Caronia and colleagues included 55 patients with FHA and 422 controls (7). They found six heterozygous variants in the *FGFR1*, *PROKR2*, *KAL1*, and *GnRHR* genes in 7 patients with FHA (7/55; 12.7%). The affected genes play fundamental roles in the ontogeny and function of the GnRH-secreting neuron. In particular, the *FGFR1* gene controls the migration and survival of GnRH neurons (44), *PROKR2* and *KAL1* genes are critical for their migration (45, 46), and *GnRHR* encodes the unique receptor that is activated by GnRH at the pituitary level (47). As reported in the previous paragraph, mutations in these genes have been associated with IHH in humans (36). All identified variants were considered pathogenic as they all were found in conserved amino acid residues and induced significant loss of function. None of these mutations were observed in the control group. All seven patients with gene mutations had secondary amenorrhea for at least 6 months and at least one factor for FHA (7). Four of these patients also reported a family history of FHA or delayed puberty, but the genetic analysis was not extended to other affected relatives to confirm the association. Two of the seven patients continued to receive long-term hormone replacement therapy (HRT), whereas the other five discontinued HRT and had their menstrual cycle resumed (7).

However, the study by Caronia and colleagues examined only a limited number of genes associated with IHH and the genetic investigation of controls was limited to the search of variants identified in patients with FHA (7). To overcome this problem, in 2020, the same group expanded the analysis to include 53 genes associated with IHH and sequenced all coding regions in 106 women with FHA and 468 controls (8).

According to the hypothesis of Caronia and colleagues (7) and with the results of their first study, Delaney and coworkers found a high frequency of heterozygous rare sequence variants associated with IHH in patients with FHA. In detail, they identified 78 heterozygous variants in 58 out of 106 patients with FHA. Two hundred control women also harbored 255 rare sequence variants. However, the frequency of these mutations was significantly higher in FHA women compared to controls. Additionally, patients with FHA were more likely to have a greater number (3 or more) of rare sequence variants compared to controls. It is also important to highlight that a greater number of mutations previously identified in patients with severe IHH phenotypes were present in FHA patients compared to controls. Furthermore, all mutations identified in women with FHA were present in heterozygosity (8). This is in line with other evidence suggesting a genotype-phenotype relationship (40–42). Therefore, patients with biallelic or di/oligogenic mutations show severe forms of GnRH deficiency resulting in IHH, while it can be hypothesized that these heterozygous mutations are not sufficient to cause IHH, but they could lower the threshold for functional inhibition of the HPO axis caused by exposure to stressful events leading to FHA. This is supported by the identification of some rare variants even in controls with regular menstrual cycles. The results of the studies conducted by Caronia and colleagues (7) and Delaney and colleagues (8) allow us to hypothesize that FHA is part of a spectrum of diseases with a genetic base (10). Indeed, as mentioned previously, Gianetti and coworkers studied the correlation between the severity of the phenotype of the HPO axis dysfunction and the severity of rare sequence variants of the *GnRHR* gene. They also included 77 patients with FHA and, among these, 3 patients presented monoallelic alterations of *GnRHR* (40). **Table 1** summarizes the rare variants of genes related to GnRH-secreting neuron function identified in patients with FHA.

**Table 1:** Rare variants of genes related to GnRH-secreting neuron function identified in patients with functional hypothalamic amenorrhea (FHA).

Gene	Description	Chromosome	Function	Identified variants in patients with FHA (Authors & Year)
*ANOS1*	Anosmin 1	chrXp22.31	GnRH neuron migration/ neurodevelopment	• His672Arg (8) • Val587Leu (8) • Ser511Tyr (8) • Val371Ile (8)
*AXL*	AXL receptor tyrosine kinase	chr19q13.2	GnRH neuron migration /neurodevelopment	• Val289Met (8) • His292Profs47 (8) • Gly517Ser (8)
*CCDC141*	Coiled-Coil Domain Containing 141	chr2q31.2	GnRH neuron migration/ neurodevelopment	• Glu876Lys (8
*CHD7*	Chromodomain helicase DNA binding protein 11	chr8q12.2	GnRH neuron migration/ neurodevelopment	• Ser244Arg (8) • Met340Val (8) • Met396Ile (8) • Arg459Cys (8) • Ser466Leu (8) • Asp728His (8) • Pro1705Gln (8) • Arg1942Trp (8) • Met2527Leu (8) • Leu2984Phe (8)
*DCC*	DCC netrin 1 receptor	chr18q21.2	GnRH neuron migration/ neurodevelopment	• Gly470Asp (8) • Asn635Ser (8) • Asp819Asn (8) • Val883Ile (8)
*DMXL2*	DMX like 2	chr15q21.2	GnRH neuron migration/ neurodevelopment	• Ile2573Val (8) • Ile1317Val (8) • Met563Val (8) • Thr476Ser (8)
*FEZF1*	Fez family zinc finger protein 1	chr7q31.32	Neurodevelopment	• Gln448Pro (8)
*FGFR1*	Fibroblast growth factor receptor 1	chr8p11.23	GnRH neuron migration/ neurodevelopment	• Arg756His (7) • Gly291Glu (7, 8)
*FLRT3*	Fibronectin like domain containing leucine rich transmembrane protein 3	chr20p12.1	GnRH neuron migration/ neurodevelopment	• Gln401Leu (8)
*GNRH1*	GnRH 1	chr8p21.2	GnRH action	• Ile48Arg (8)
*GNRHR*	GnRH receptor	chr4q13.2	GnRH action	• Arg262Gln (7, 8, 40) • Ser168Agr (8) • Ser168Ala (40) • Gln106Arg (8, 40
*HESX1*	Homeobox gene expressed in stem cells 1	chr3p14.3	Neurodevelopment	• Val129Ile (8)
*KAL1*	Kallman 1	chrXp22.31	GnRH neuron migration/ neurodevelopment	• Asp1206Tyr (7)
*KL*	Klotho	chr13q13.1	Neurodevelopment	• Arg751Gly (8) • Val845Gly (8)
*KLB*	Klotho beta	chr4p14	GnRH neuron migration/ neurodevelopment	• Ala169Thr (8) • Lys815Glu (8) • Gly908Val (8) • Val1042Ile (8)
*LEPR*	Leptin receptor	chr1p31.3	Neuroendocrine	• Val754Met (8)
*LHX3*	LIM homeobox 3	chr9q34.3	Neurodevelopment	• Gly317Ser (8) • Arg315Pro (8)
*NR0B1*	Nuclear receptor subfamily 0, group B, member 1	chrXp21.2	Neuroendocrine	• Ser412Gly (8)
*OTUD4*	OUT domain containing protein 4	chr4q31.21	Neurodevelopment	• Pro933Arg (8)
*PCSK1*	Proprotein convertase subtilisin/kexin type 1	chr5q15	Neurodevelopment	• Thr640Ala (8)
*PNPLA6*	Patatin-like phspholipase domain-containing protein 6	chr19p13.2	Neurodevelopment	• Gly1329Arg (8)
*POLR3B*	Polymerase III RNA subunit B	chr12q23.3	Neurodevelopment	• Met415Thr (8) • Lys721* (8) • Arg978Cys (8)
*PROK2*	Prokineticin 2	chr3p13	GnRH neuron migration/neurodevelopment	• Met61Val (8)
*PROKR2*	Prokineticin receptor 2	chr20p12.3	GnRH neuron migration/neurodevelopment	• Leu173Arg (7) • Thr340Ser (8) • Met111Arg (8) • Arg85His (7, 8)
*PROP1*	Homeobox protein prophet of Pit-1	chr5q35.3	Neurodevelopment	• Ala142Val (8)
*RAB3GAP1*	RAB3 GTPase activating non-catalytic protein subunit 1	chr2q21.3	Neurodevelopment	• Arg336Cys (8) • Arg954His (8)
*RAB3GAP2*	RAB3 GTPase activating non-catalytic protein subunit 2	chr1q41	Neurodevelopment	• Leu1331Ile (8) • Asp1206Tyr (8) • Leu764Phe (8) • Pro527Leu (8) • Arg420Cys (8)
*SEMA3A*	Semapthorin 3A	chr7q21.11	GnRH neuron migration/ neurodevelopment	• Asn153Ser (8)
*SEMA3E*	Semapthorin 3E	chr7q21.11 chr7q21.11	GnRH neuron migration/neurodevelopment	• Asp580Asn (8) • Pro171Ser (8)
*SOX2*	Sex determining region Y-box 2	chr3q26.33	GnRH neuron migration/ neurodevelopment	• Gly22Ser (8)
*SPRY4*	Sprouty drosophila homolog of 4	chr5q31.3	Neurodevelopment	• Ser241Tyr (8) • Cys209Tyr (8) • Gly92Val (8)
*SRA1*	Steroid receptor RNA activator 1	chr5q31.3	Steroid activity	• 110AspfsTer25 (8)
*TACR3*	Tachykinin receptor 3	chr4q24	GnRH secretion	• His248Arg (8)
*WDR11*	WD Repeat-Containing protein 11	chr10q26.21	GnRH neuron migration/ neurodevelopment	• Val6Met (8)

## 5 Epigenetic modifications

Epigenetics is a field of study that explains how environmental factors can modify gene expression without changing the DNA sequence. It involves processes that regulate gene expression without altering the structure of chromatin (48). The three main epigenetic mechanisms that regulate gene activity regulation are DNA methylation, histone modifications, and non-coding RNAs. Recent research has highlighted the significance of epigenetic modifications in the pathogenesis of various human diseases. For instance, epigenetic changes could potentially represent the mechanism by which stressful events can triggers FHA in women with a susceptibility genotype (10).

Over the last decade, the significance of epigenetic factors in the expression of genes involved in GnRH-neuron function has increased. *In vitro* studies have shown that the *GnRH* gene responds to external stimuli by modifying chromatin in mature GnRH neurons as well (49). Studies on animal models have demonstrated that nutritional changes can affect the expression of *KISS1* (the gene encoding kisspeptin) during puberty through epigenetic mechanisms and is regulated by sirtuin 1 (SIRT1) (50). When undenutrition occurs, the activity of SIRT1 increases, leading to prolonged *KISS1* suppression and delayed onset of puberty in negative energy balance conditions (50).

Altered patterns of DNA methylation have been found in patients with anorexia nervosa (AN) in relation to several genes, including *synuclein alpha*, *dopamine transporter*, *dopamine receptor D2*, *oxytocin receptor*, and *LEP* (51–53). Women with AN have been found to exhibit lower *LEP* and *LEPR* methylation compared to controls. Interestingly, women with lower DNA methylation of the *LEP* gene have shown full recovery after psychotherapeutic treatment, and a significant hypermethylation of the *LEP* gene during treatment (52). These results suggest that *LEP* methylation could serve as a predictor to identify patients with a higher chance of recovery after treatment (52).

Ghrelin, along with leptin, plays an important role in regulating appetite and energy expenditure. By activating its receptor (growth hormone secretagogue receptor, GHS-R1a), ghrelin induces an orexigenic state, which facilitates food intake (54). GHS-R1a promoter methylation was found to be increased in patients with AN compared to healthy controls (53). Hypermethylation of the *GHS-R1a* promoter might be a consequence of acute undernutrition, inhibiting the expression of the *GHS-R1a* gene. This is consistent with several studies that have reported a dysregulation of ghrelin signaling pathway in patients with AN, which have increased ghrelin levels, suggesting a condition of ghrelin resistance (55).

Recent studies have also revealed that microRNAs (miRNAs) may play a significant role in regulating GnRH secretion and may be implicated in the pathophysiology of FHA (10, 56). miRNAs are small non-coding RNA molecules, which play a critical role in regulating gene expression by binding to messenger RNAs (mRNAs) and inhibiting their translation or promoting their degradation. Some miRNAs (miR- 132, miR-212, miR-361-3p) have been reported to be induced by GnRH (57, 58) and play a role in gonadotropin pathways. In addition, specific miRNAs, such as miR-34c, have been linked to the regulation of stress-related pathways (59), which are critical in FHA. Although microRNAs are not yet in clinical use, these findings open new research horizons to identify the involvement of miRNAs in FHA and their possible use as peripheral biomarkers to monitor effects of treatment in FHA (10).

## 6 Is functional hypogonadotropic hypogonadism the male equivalent of functional hypothalamic amenorrhea?

There have been only a few studies that have examined the male phenotypic counterpart of FHA. Previous research has found that men who engage in strenuous physical exercise (60–68) or undergo periods of caloric restriction (69–71) may experience dysregulation of their hypothalamic-pituitary-testicular (HPT) axis.

In 1986, MacConnie and colleagues conducted one of the first studies investigating the integrity of hypothalamic-pituitary-gonadal (HPG) axis in athletes. The authors found that six highly trained marathon runners (who were running 125 to 200 km per week) had decreased LH pulse frequency and amplitude (every-20-minute sampling for 8 hours) compared to healthy controls (62). Several studies have shown that soldiers who are exposed to prolonged caloric restriction, high energy expenditure, and disrupted sleep may experience a reduction in their testosterone levels (72, 73). In 2019, Dwyer and coworkers conducted a study on 18 young men who presented for evaluation of hypogonadism secondary to excessive exercise or weight loss and investigated their biochemical and genetic profiles (74). The results showed patients had significantly lower body mass index (BMI), testosterone, and LH compared to controls. Men with functional HH had a lower LH pulse amplitude then controls. So far, no studies have investigated the impact of excessive physical exercise or caloric restriction on spermatogenesis. Only 3 of the 10 patients who participated in the study underwent a semen analysis that showed a normal sperm count. However, serum inhibin B levels were significantly lower in patients compared to controls (74). Inhibin B is a glycoprotein hormone produced by Sertoli cells that inhibits FSH secretion and stimulates testosterone secretion by Leydig cells (75). It is, therefore, an essential marker of spermatogenesis.

Previous studies have not provided clear information on whether the decrease in testosterone levels in men due to excessive strenous exercise or caloric restriction was associated with clinical symptoms of hypogonadism. However, a study conducted by Dwyer and colleagues found that 9 out of the 10 patients examined presented low libido, which was associated with erectile dysfunctions in two patients and with reduction of potency/training performance in six patients (74). It is important to note that two of the 10 patients included had a family history of FHA and two patients had two rare genetic variants in genes related to GnRH deficiency, *Chromodomain Helicase DNA-binding Protein 7* (*CHD7*) and *NMDA Receptor Synaptonuclear Signaling and Neuronal Migration Factor* (*NSMF*) (74). As reported in paragraph 4.1, in both men and women, mutations in genes related to GnRH can lead to a wide spectrum of reproductive phenotypes. Men with biallelic partial loss-of-function mutations in GnRHR may experience delayed puberty, congenital HH (CHH), and reversal and relapse of CHH (40, 76, 77). Hietamaki and colleagues described the phenotypic and genetic characteristics of two monozygotic twin brothers with stalled puberty. They had heterozygous mutations in the *GnRHR* gene and, interestingly, the one with higher BMI had lower testosterone levels. This finding suggests that excess adipose tissue may suppress the HPT axis in patients with a partial form of gonadotropin deficiency (42). Metabolic hypogonadism is a well-characterized condition in men (78, 79), although a similar condition has recently been proposed in women, as “female obesity-related secondary hypogonadism” (80). In men, obesity can significantly compromise the gonadal function through different mechanisms, including disruption of the hormonal milieu, induction of systemic low-grade inflammation, and increased oxidative stress with consequent alterations of conventional and biofunctional sperm parameters (78). Obesity also causes epigenetic changes that can be transferred to offspring (78). In men, changes in the normal methylation pattern of the genes *H19*/*insulin-like growth factor 2* and other imprinted genes have been associated with infertility and the risk of transmitting epigenetic abnormalities to offspring (81).

Reproduction is a complex process influenced by environmental, social, neuronal, endocrine, and metabolic factors. Unlike women, men appear to be more resistant to stress-induced alterations in gonadal function. In women, FHA is considered an adaptive response of the female body to stress. This adaptation is thought to be the body’s ability to priorize energy allocation to vital physiological functions over reproduction and pregnancy when energy intake is insufficient (82). It is less clear whether functional HH may also represent a similar adaptive response to conserve energy in men when energy is limited. However, because reproductive function is not essential for survival and requires a large amount of energy, it is biologically plausible that stressors may interfere in both sexes. Certainly, in women, FHA provides a clear model for understanding how stress reduces the function of the HPG axis. Unlike women who experience the evident symptom of amenorrhea, men show less obvious clinical manifestations, although similar mechanisms cannot be ruled out. For this reason, further studies are needed to examine the hormonal and genetic profile of men undergoing strenuous physical exercise, weight loss, or prolonged psychological stress, to identify a potential role in male sexual and reproductive health.

## 7 Final remarks

**Figure 1:**
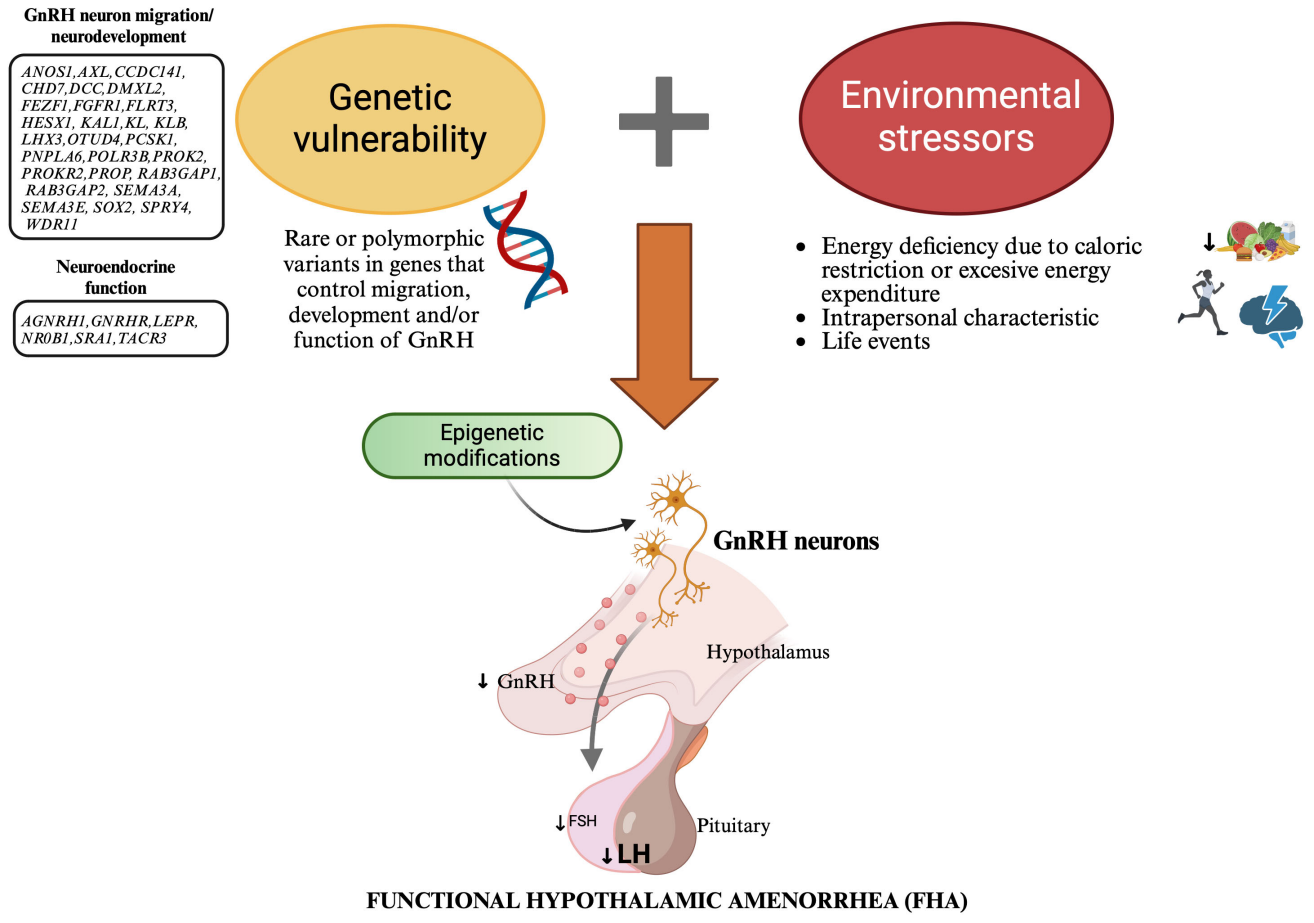
New model explaining the etiopathogenesis of functional hypothalamic amenorrhea: a combination of genetic predisposition, environmental factors, and epigenetic changes. This figure illustrates a new model for the etiopathogenesis of functional hypothalamic amenorrhea (FHA), highlighting the interplay between genetic predisposition, environmental factors, and epigenetic changes. Genetic vulnerability: Rare genetic variants in genes associated with idiopathic hypogonadotropic hypogonadism (IHH), which control the migration, development and/or function of GnRH neurons, predispose some women to FHA by impairing GnRH neuron function. Environmental factors: Energy deficiency due to caloric restriction or excessive energy expenditure, intrapersonal characteristics, and life events act as stressors that disrupt normal GnRH secretion. Stressors have a particularly significant impact in genetically predisposed people, leading to decreased LH levels and subsequent menstrual irregularities. Epigenetic changes: Environmental stressors induce epigenetic modifications, such as DNA methylation, histone modification, and miRNA regulation. These changes alter gene expression without changing the DNA sequence, resulting in long-lasting hypothalamic dysfunction. This model highlihghts the importance of personalized approaches in the diagnosis, treatment, and prevention of FHA. GnRH, Gonadotropin Releasing Hormone; FSH, follicle stimulating hormone (FSH); LH, luteinizing hormone.

Recent evidence has highlighted a genetic predisposition to FHA that could explain inter-individual variability in response to stress. Indeed, rare variants in genes associated with IHH have been identified in women with FHA, suggesting that these mutations may contribute to increasing women’s susceptibility to stressor triggers. FHA appears today as a complex disease resulting from the combination of genetic predisposition, environmental factors, and epigenetic changes (**Figure 1**). The identification of rare genetic variants associated with FHA holds promise for advancing our understanding of the condition and developing personalized approaches to this disease, ultimately improving both short-term management and long-term health consequences for affected women.

Further studies are needed to examine better rare variants in genes associated with IHH in larger cohorts of women with FHA possibly in multicenter studies. When designing new research, it may be interesting to match healthy controls to cases based on exposure to risk factors. Genetic investigation could be very useful for patients experiencing amenorrhea, where stress, as a trigger, may not be strong enough to justify FHA, or even in cases where the menstrual cycle fails to resume despite stress factors being resolved. In such situations, there may be an underlying genetic predisposition that needs to be identified.

This emerging genetic predisposition to FHA may be even more complex. Indeed, previous studies have shown genetic overlap not only with genes linked to IHH but also with other psychological disorders, such as anxiety and mood disorders (10). Therefore, exploring rare genetic variants of genes involved in appetite control, energy homeostasis, and stress response pathways could be highly interesting.

Finally, the genetic background of FHA suggests the existence of a male equivalent. To date, little is known about the existence of a “functional hypogonadotropic hypogonadism” and its possible short- and long-term consequences in men. Indeed, there have been few studies on the clinical presentation and hormonal imbalance in men who have undergone caloric restriction, strenuous physical activity, and psychological stress. To date, only one small sized study has examined the genetic profile of these patients. Despite the paucity of data, preliminary findings suggest the existence of a condition parallel to FHA in men, thus warranting further investigation.
